# Image Features of Magnetic Resonance Imaging under the Deep Learning Algorithm in the Diagnosis and Nursing of Malignant Tumors

**DOI:** 10.1155/2021/1104611

**Published:** 2021-08-30

**Authors:** Lifang Sun, Xi Hu, Yutao Liu, Hengyu Cai

**Affiliations:** ^1^Department of Hematology-Oncology, The Third Hospital of Jilin University, Changchun 130033, Jilin, China; ^2^Department of Gynaecology, The Third Hospital of Jilin University, Changchun 130033, Jilin, China; ^3^Department of Central Sterile Supply, The Third Hospital of Jilin University, Changchun 130033, Jilin, China

## Abstract

In order to explore the effect of convolutional neural network (CNN) algorithm based on deep learning on magnetic resonance imaging (MRI) images of brain tumor patients and evaluate the practical value of MRI image features based on deep learning algorithm in the clinical diagnosis and nursing of malignant tumors, in this study, a brain tumor MRI image model based on the CNN algorithm was constructed, and 80 patients with brain tumors were selected as the research objects. They were divided into an experimental group (CNN algorithm) and a control group (traditional algorithm). The patients were nursed in the whole process. The macroscopic characteristics and imaging index of the MRI image and anxiety of patients in two groups were compared and analyzed. In addition, the image quality after nursing was checked. The results of the study revealed that the MRI characteristics of brain tumors based on CNN algorithm were clearer and more accurate in the fluid-attenuated inversion recovery (FLAIR), MRI T1, T1c, and T2; in terms of accuracy, sensitivity, and specificity, the mean value was 0.83, 0.84, and 0.83, which had obvious advantages compared with the traditional algorithm (*P* < 0.05). The patients in the nursing group showed lower depression scores and better MRI images in contrast to the control group (*P* < 0.05). Therefore, the deep learning algorithm can further accurately analyze the MRI image characteristics of brain tumor patients on the basis of conventional algorithms, showing high sensitivity and specificity, which improved the application value of MRI image characteristics in the diagnosis of malignant tumors. In addition, effective nursing for patients undergoing analysis and diagnosis on brain tumor MRI image characteristics can alleviate the patient's anxiety and ensure that high-quality MRI images were obtained after the examination.

## 1. Introduction

Brain tumor is a cell that grows abnormally in brain tissue, causing serious damage in the human body and posing a threat to the health of patients [1]. Clinically, brain tumor is divided into primary brain tumor and secondary brain tumor. The secondary brain tumor is a kind of malignant tumors that originates outside the brain and spreads from other parts of the patient's body and gradually transitions into the brain [2]. Glioma is one of the most frequently found malignant brain tumors and grows abnormally fast and in the diffuse form, which can seriously threaten the patient's central nervous system [3].

Nowadays, under the research and exploration of clinical tumor diagnosis and treatment technology, computerized tomography (CT), magnetic resonance imaging (MRI), and other imaging technologies have become the main force in medical image analysis. The detection rate of malignant tumors has also generally increased, which allows the harm of tumors to the human body to be controlled, and the survival period of patients with malignant tumors has begun to increase [4]. Among them, MRI imaging technology has become a common method for the diagnosis and treatment of brain tumors. The application of MRI technology makes various imaging parameters produce various different modal maps, which bring rich and colorful image characteristics and information for the diagnosis and treatment of brain tumors [5]. In the past, the conventional artificial segmentation semantic model was very time-consuming and was interfered by the clinical experience and subjective judgment of the attending physician. Deep learning, especially the application of convolutional neural network (CNN) algorithm, enabled image recognition and feature analysis in the computer field. The research and exploration have been further developed. In addition, the CNN algorithm has shown its characteristics beyond other algorithms in natural image segmentation, which brings a good opportunity for the diagnosis and feature analysis of brain tumor images [6]. Jeffrey's study and other related research studies also showed that deep learning algorithm technology showed excellent analysis performance on MRI images, which promoted the detection rate of breast cancer, brain tumor, and other malignant tumors [7].

Targeted therapy is a research hotspot in the field of tumor diagnosis and treatment, showing the characteristics of small trauma and low toxicity. However, the effect of the MRI imaging technology on the observation and detection of targeted controlled release of nanoformulations is not ideal, and it needs to be combined with other technologies for in-depth analysis [8]. In recent years, with the in-depth study of convolutional neural network algorithm of deep learning by experts, this technology has been widely used in clinical medical images so that image analysis technology based on CNN algorithm has made remarkable achievements in the field of MRI image feature analysis. Because the scanning time of this technology is relatively long for patients, it needs the high cooperation of the subjects, so it puts forward high requirements for patients. Then, to reduce the anxiety of patients during the examination and show a good degree of cooperation, high requirements are placed on the nursing technology in magnetic resonance detection. It has to summarize the nursing methods in the development and application of new technologies, so as to validate the effectiveness. Based on this, a brain tumor MRI image model based on the CNN algorithm was constructed in this study, the impacts of the algorithm on MRI images of brain tumor patients were analyzed, and the practical value of MRI image characteristics based on deep learning algorithms in the clinical diagnosis and nursing of brain tumors was evaluated. In this way, it aimed to provide a good theoretical basis for the clinical application of MRI image characteristics based on deep learning algorithms in the diagnosis and nursing of malignant tumors.

## 2. Materials and Methods

### 2.1. Research Objects

80 patients who were admitted in the hospital from April 2018 to April 2020 were selected for brain tumor-specific detection, including 32 males and 48 females, for MRI imaging examination. The age range was 22–81 years, with an average age of 52 years.

The inclusion criteria of this study were patients who had not received surgery, radiotherapy, or chemotherapy before the experiment, patients with complete clinical basic data, and patients with clear preoperative MRI images; the exclusion criteria included patients with severe brain damage, hypersensitivity to contrast agents, other malignant tumors, and poor-quality MRI images with many artifacts.

This study had been approved by the ethics committee of the hospital, and the patients and their families signed the informed consent forms.

### 2.2. Grouping of Research Objects

All research objects underwent MRI scans and were numbered if the criteria were met. According to the random number table generated by the computer, the patients were randomly divided into 2 groups at a ratio of 1 : 1, the experimental group and the control group, with 40 cases in each group. Patients in the experimental group received CNN-based MRI image analysis, and patients in the control group received conventional MRI image analysis methods.

### 2.3. MRI

Under the research and exploration of clinical tumor diagnosis and treatment technology, imaging technologies such as CT and MRI have become the main force in medical image analysis. Compared with CT, MRI shows high accuracy and sensitivity in imaging various tissues of the human brain. MRI imaging technology has become a common method for the diagnosis and treatment of brain tumors [9]. In imaging, the modalities obtained after the analysis of different parameters are different, and the differences between them are relatively large so that the final results presented show different information. In clinical studies of brain tumors, researchers often diagnose and treat tumors through the four modal sequences of MRI fluid-attenuated inversion recovery (FLAIR), MRI T1, T1c, and T2 [10]. Among them, the FLAIR modal image can easily identify the difference between the diseased module and the normal module, but it is more difficult to project the brain tissue structure. The modal image of MRI T1 shows that the grayscale of the lesion area and its surrounding structures are slightly different; the grayscale of each part of the brain displayed by the modal image is relatively similar, and there is no obvious difference between the lesion area and the normal part. The modal image of MRI T1c can show the tissue framework of each area of the brain so that the diseased part and the normal part can be distinguished well, and the difference is large. The image of MRI T2 shows the overall contrast for the grayscale of the diseased part and the normal brain area [11].

### 2.4. Analysis of Brain Tumor MRI Image Modeling Based on Conventional Algorithms

The expressive ability of the network is complicated with the increase of the true network depth. In the neural convolutional network, the deep convolutional layer generally has more complicated and abstract semantic features. After sampling by the pooling layer, the detailed information of low-level features such as various edges or positions may be lost, but the lost language information has great application value in the MRI technology.

**Figure 1:**
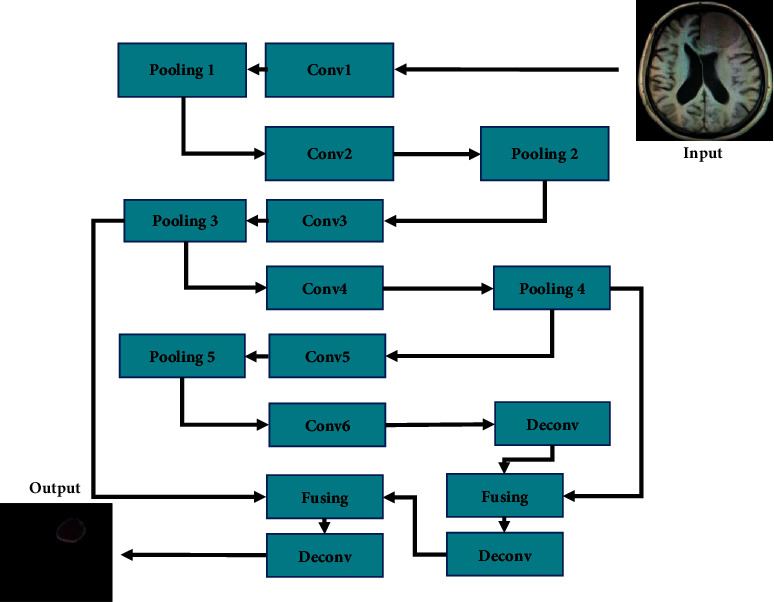
The diagram for the CNN structure of brain tumor MRI.

Figure 1 describes the CNN structure of the conventional MRI brain tumor. It illustrated that information sampling was performed on the last layer of the network to combine it with the information of the middle layer so that it can have good performance. Due to the blurry edges of the tumor and the complicated internal tissues, various modal expression information was usually composed of the same layer of the input four modal MRI images to form a four-channel image. The input image of the conventional MRI algorithm was a four-channel image composed of slices in the same layer according to the four modal sequences of FLAIR, T1, T1c, and T2. The final output channel number was 5, corresponding to 5 categories of the brain tumor.

### 2.5. Brain Tumor MRI Image Modeling Based on CNN Algorithm

Using deep learning algorithms to automatically segment brain tumor MRI images has great application value in clinical tumor diagnosis and can greatly reduce the degree of dependence on the subjective judgment of doctors.

**Figure 2:**
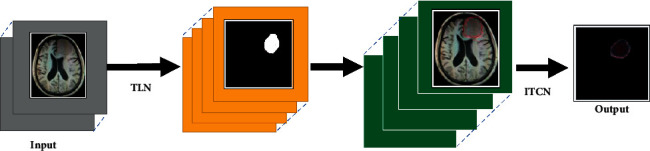
The MRI brain tumor convolutional network structure based on the CNN algorithm.

Figure 2 depicts the MRI brain tumor convolutional network structure based on the CNN algorithm. This neural convolutional network selected four-channel images combined by the same slice through the four modal sequences of magnetic resonance FLAIR, T1, T1c, and T2 and then outputted the image of the diseased area. This process included two operating procedures. In the first procedure, the structure can accurately find and mark the location of brain tumors through semantic segmentation technology. Then, on the basis of the deep learning algorithm, four subregions with different positions were segmented out of the localized tumor parts. In the second process, it only needed to process the tumor site marked in the previous process to finely segment the internal area of the brain tumor, and no postprocessing was required to shorten the time required for forward calculations. The small networks in the neural convolutional network can be divided into two types: one is the tumor localization network (TLN), and the other is the intratumor classification network (ITCN). The MRI brain tumor convolutional network structure based on the CNN algorithm is shown in Figure 2.

### 2.6. Evaluation Index and Diagnosis Method of the Brain Tumor MRI Image Based on CNN Algorithm

Evaluation indicators such as accuracy, sensitivity, specificity, and area under the curve (AUC) were used to measure the advantages and disadvantages of the algorithm. In this study, the release of the diagnostic targeted drug in the MRI image was correctly defined as *a*, the release error was defined as *b*, the actual target drug release rate was correctly defined as *c*, and the release error was defined as *d*. Accuracy referred to the proportion of correct MRI image measurement samples in the total number of samples, as shown in equation (1). Sensitivity was also called recall rate, which represented the proportion of correct samples determined by MRI images in all correct samples actually targeted for release, as shown in equation (2). Specificity referred to the proportion of the target release error samples judged by MRI in all actual target release error samples, as given in equation (3). AUC referred to the area of the part enclosed by the curve and the coordinate axis, which should not be greater than 1, as shown in equation (4).

(1) $$\begin{array}{c} \text{Accuracy}=\frac{a+d}{a+b+c+d}\times 100\%, \end{array}$$

(2) $$\begin{array}{c} \text{sensitivity}=\frac{a}{a+c}\times 100\%, \end{array}$$

(3) $$\begin{array}{c} \text{specificity}=\frac{a}{a+b}\times 100\%, \end{array}$$

(4) $$\begin{array}{c} \text{AUC}=\frac{d}{c+d}\times 100\%. \end{array}$$

### 2.7. Nursing Method

The patients in the experimental group were randomly divided into a nursing group (20 cases) and a control group (20 cases). The nursing group adopted the whole nursing method, and the control group did not carry out nursing. The details of the nursing method were as follows.

Before the examination, the patient was required to fast for at least 3.5 hours; the doctor had to check the application form to ensure that the metal foreign body, including the pacemaker, was removed from the patient; the patient was informed about the examination process and requirements and other precautions (there were lots of scanning sequences for the examination method used in this study, the scanning time was 20 minutes, and the patient was required to cooperate in the apnea scan strictly and the breath was required to slow down); the patient was informed about the adverse reactions such as dizziness and nausea, which should be handled in time to make them in a relaxed state; the patient was trained apnea exercises; and the patient's own weight had to be checked to prepare the corresponding drug dosage.

During the examination, the patient was required to remain supine with his hands raised above his head and wear earphones; the patient was required to brake and was informed that changes in the position would affect the quality of the scan. The radio frequency (RF) was uniformly placed on the patient's abdomen, and the body surface coil was fixed at the edge of the bed. The center of the coil was aligned with the midpoint of the umbilical line and the xiphoid process. The contrast agent was used: injection dose of gadolinium-ethoxybenzyl-diethylenetriamine pentaacetic acid (Gd-EOB -DTPA) was 0.01 L (0.1g/L), and the medicament in the syringe was 0.1 g/L. In addition, the amount of the contrast agent used was calculated according to body weight: based on the adult's need to inject 0.1 mL of medicament per 1 kg of body weight, and the high-pressure injection device was set. The intravenous indwelling needle was connected to the high-pressure syringe, and physiological saline was injected manually to observe whether there was leakage. The patient was instructed to learn how to use the alarm device, how to cooperate with the apnea scan according to the password, and how to keep the brake during the entire examination.

After the examination, the patient had to be instructed to stand up slowly to avoid dizziness and other reactions. Then, the patient had to sit quietly in the resting area for half an hour. After it was confirmed that there was no abnormality, the needle was removed, and the injection part was pressed until no blood leaked.

### 2.8. Evaluation Indicators

During the examination, the patient's anxiety level was measured and scored using the Hamilton Anxiety Questionnaire (HAMA) [12]. The items in the HAMA were all scored at 0–3. The evaluation criteria were as follows: no anxiety scored 0 points, mild anxiety scored 1 point, 2 points were given for moderate anxiety, and 3 points were defined for severe anxiety.

The quality of MRI images was evaluated by three doctors with many years of clinical medical imaging experience. The scoring standards were divided into poor, medium, and excellent. Among them, if the image quality was with many shadows, blurred organizational structure boundaries, and it was difficult to recognize the content, the image was evaluated as poor; if the image showed light shadows but did not affect the evaluation of doctors, the image quality was rated as medium; and if the image showed clear tissues without artifacts and the boundaries on each edge were clear, it could be rated as excellent.

### 2.9. Statistical Analysis

SPSS 24.0 software was adopted for statistical analysis, expressed as mean standard deviation (*x*‾ ± *s*). The statistical data were analyzed by *t*-test, and the test level was *α* = 0.05. *P* < 0.05 indicated that the difference was statistically significant.

## 3. Results

### 3.1. Analysis on Macroscopic Characteristics of MRI Images of Patients with the Brain Tumor

**Figure 3:**
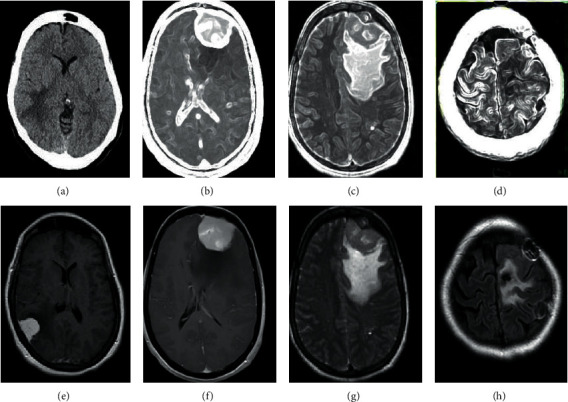
The MRI image of a 48-year-old female patient with the brain tumor. (a–d) FLAIR, T1, T1c, and T2 modal images processed by conventional MRI image algorithms, respectively. (e–h) FLAIR, T1, T1c, and T2 modal images processed by CNN algorithm, respectively.

Figure 3 shows an MRI image of a 48-year-old female patient with the brain tumor. Compared with the uniform signal of the normal brain, the MRI image signal of brain tumor patients was unevenly distributed and diffuse. Some tumors had envelopes, some were small nodules, and some were large masses. Figures 3(a)–3(d) show the four modalities of conventional MRI images, and Figures 3(e)–3(h) show the four modalities of MRI images processed based on the CNN algorithm, followed by FLAIR, T1, T1c, and T2. The results in Figure 3 showed that the deep learning algorithm can quickly and accurately mark and segment each brain tumor area and the internal tissue structure of the tumor with different sizes and appearances, and each image was close to the real result. In addition, the MRI image characteristics of the brain tumor based on the CNN algorithm were more accurate and clearer than the MRI image features of the conventional algorithm, which can accurately identify the location of the patient's brain tumor. Such results indicated that the deep learning algorithm can accurately identify and segment brain tumors of various sizes and states and the corresponding brain lesions, and the image results of each mode were very close to the truth labels.

### 3.2. The Imaging Index of MRI Image Analysis Algorithm Based on the CNN

The accuracy, sensitivity, specificity, and AUC of the two algorithms on MRI images were compared, and the following results were obtained and illustrated as follows.

**Figure 4:**
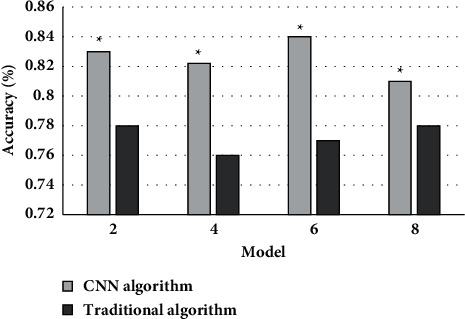
Comparison of the accuracy of the two algorithms on MRI images. Note: ^∗^suggested that the difference was statistically obvious in contrast to the conventional algorithm (*P* < 0.05).

Figure 4 shows the comparison of the accuracy of the two algorithms on MRI images. It could be concluded that the average accuracy of MRI images based on CNN and traditional algorithms was 0.83 and 0.77, the MRI image features based on CNN algorithm had obvious advantages compared with conventional algorithms, and the difference between the two was statistically obvious (*P* < 0.05).

**Figure 5:**
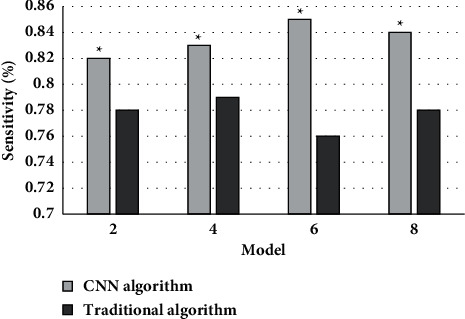
Comparison of the sensitivity of the two algorithms on MRI images. Note: ^∗^suggested that the difference was statistically obvious in contrast to the conventional algorithm (*P* < 0.05).

Figure 5 shows the comparison of the sensitivity of the two algorithms on MRI images. It could be concluded that the average sensitivity of MRI images based on CNN and traditional algorithms was 0.84 and 0.78, the MRI image features based on CNN algorithm had obvious advantages compared with conventional algorithms, and the difference between the two was statistically obvious (*P* < 0.05).

**Figure 6:**
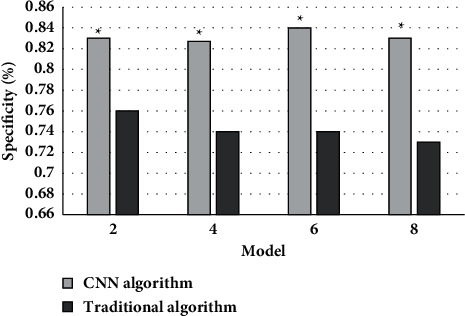
Comparison of the specificity of the two algorithms on MRI images. Note: ^∗^suggested that the difference was statistically obvious in contrast to the conventional algorithm (*P* < 0.05).

Figure 6 shows the comparison of the specificity of the two algorithms on MRI images. It could be concluded that the average specificity of MRI images based on CNN and traditional algorithms was 0.83 and 0.8, the MRI image features based on CNN algorithm had obvious advantages compared with conventional algorithms, and the difference between the two was statistically obvious (*P* < 0.05).

**Figure 7:**
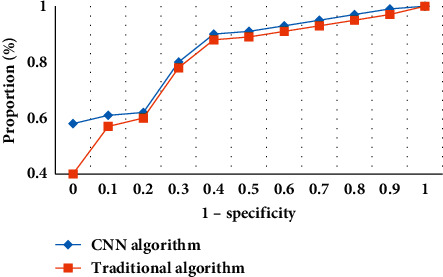
Comparison of the AUC of MRI images between the two algorithms.

Figure 7 illustrates the comparison of the AUC of MRI images between the two algorithms. It revealed that, in terms of the AUC, the MRI image features based on the CNN algorithm showed obvious advantages compared to the MRI image features of the conventional algorithm.

From the above results, it could be concluded that compared with conventional algorithms, CNN-based MRI image algorithms showed obvious advantages in accuracy, sensitivity, specificity, and AUC, showing statistically observable differences (*P* < 0.05). These comparison results suggested that deep learning algorithms can further accurately segment the MRI images of liver cancer patients on the basis of conventional algorithms, thereby improving the application value of MRI images.

### 3.3. Comparison of HAMA Scores between the Nursing Group and Nonnursing Group

**Figure 8:**
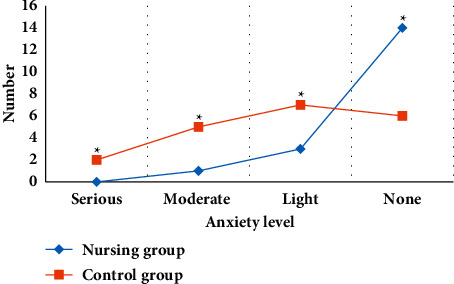
Anxiety of patients in the nursing group and nonnursing group. Note: ^∗^suggested *P* < 0.05 in contrast to the level in the nursing group.

The HAMA score scale is an index for scoring the degree of anxiety in patients with the brain tumor after surgery. The HAMA score of the nursing group was dramatically better than that of the nonnursing group. Compared with the nonnursing group, no patients who received the whole nursing group had severe anxiety, and there were 14 patients without anxiety. The difference between the two groups was statistically significant (*P* < 0.05) (as shown in Figure 8).

### 3.4. Evaluation of MRI Image Quality between the Two Groups

**Figure 9:**
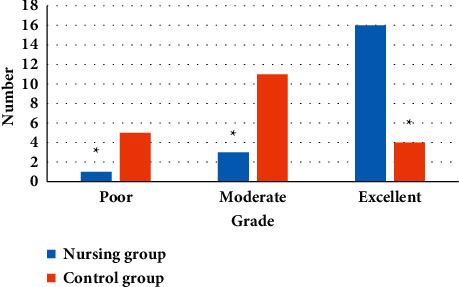
MRI image quality score of patients in the nursing group and nonnursing group. Note: ^∗^suggested *P* < 0.05 in contrast to the level in the nursing group.

The image quality of the two groups of patients was evaluated, and the comparison result shown in Figure 9 was obtained. The figure illustrated that the image score of the nursing group was much higher in contrast to that of the control group, and the difference between the two was statistically great (*P* < 0.05). In the nonnursing group, there were 5 patients whose image quality could not be assessed, diagnosed, and treated. After the whole course of nursing these 5 patients, their image quality was greatly improved so that clinicians made accurate judgments smoothly.

## 4. Discussion

Brain tumor seriously damages the life and health of patients, so timely and effective early diagnosis, prevention, and control are very important [13]. MRI technology has become a common method for the diagnosis and treatment of brain tumors. The application of MRI technology makes various imaging parameters produce various different modal maps, which bring rich and colorful image characteristics to the diagnosis and treatment of brain tumors. The neural convolutional network structure has the ability to quickly segment the complete brain lesions of patients with tumors and can well handle the pixel problems of the internal tissues of the brain tumor and normal structure tissues [14].

Imaging technologies such as CT and MRI have become the main force in medical image analysis. Among them, MRI is more sensitive than CT in imaging various tissues of the human brain, which makes the application of MRI technology to diagnose and treat brain tumors as the main way [15]. The results of the analysis of macroscopic characteristics of MRI images showed that the MRI image characteristics of the brain tumor based on the CNN algorithm were more accurate and clearer than the MRI image features of the conventional algorithm, which can accurately identify the location of the patient's brain tumor. Such results indicated that the deep learning algorithm can accurately identify and segment brain tumors of various sizes and states and the corresponding brain lesions, and the image results of each mode were very close to the truth labels.

The emergence of deep learning algorithms, especially the emergence of CNN algorithms, has made great progress in the task of image identification and diagnosis in the field of clinical imaging, accelerating the rapid development in this field [16]. Furthermore, the wide application of these algorithms in the field of medical imaging makes the CNN algorithm present obvious advantages in image analysis and diagnosis [17]. The results of the imaging index analysis of CNN-based MRI image features found that the CNN-based MRI image algorithm showed obvious advantages in accuracy, sensitivity, specificity, and AUC, and the differences were statistically great (*P* < 0.05), which was consistent with the research results of Chen et al. [18]. These comparison results suggested that deep learning algorithms can further accurately segment the MRI images of brain cancer patients on the basis of conventional algorithms, thereby improving the application value of MRI images.

Nursing of patients in the nursing group and nonnursing group during the entire examination process was the guarantee for the smooth completion of the examination and the acquisition of high-quality imaging data. Based on the study of MRI image features based on the CNN algorithm, it was concluded that the MRI brain tumor-specific inspection technology was a full-nursing method. The results of a randomized controlled study showed that this effective nursing method can reduce the anxiety of patients and was of great significance in ensuring that high-quality images were obtained after the examination.

## 5. Conclusion

A CNN-based image analysis algorithm was designed and applied to the analysis and diagnosis of MRI image characteristics of patients with cerebral aneurysm, so as to explore the diagnosis and nursing of MRI image features processed by the CNN-based image segmentation algorithm on malignant brain tumors. The results of the study revealed that the MRI characteristics of brain tumors based on CNN algorithm were clearer and more accurate in the fluid-attenuated inversion recovery (FLAIR), MRI T1, T1c, and T2; in terms of accuracy, sensitivity, and specificity, the mean value was 0.83, 0.84, and 0.83, which had obvious advantages compared with the traditional algorithm (*P* < 0.05). Compared with the control group, the nursing group showed lower depression score and better MRI images. Therefore, the deep learning algorithm can further accurately analyze the MRI image characteristics of brain tumor patients on the basis of conventional algorithms, with high sensitivity and strong specificity, which improved the application value of MRI image characteristics in the diagnosis of malignant tumors. Effective nursing for patients undergoing brain tumor MRI image feature analysis and diagnosis can reduce patient anxiety and ensure that high-quality MRI images were obtained after the examination. The shortcoming of this study was that the research results would have certain errors and biases compared with the overall population due to the small sample size, so it was difficult to promote. Therefore, it will be necessary to expand the sample size and further explore and discuss large samples in future studies, so as to overcome shortcomings and promote the conclusions.

## Data Availability

The data used to support the findings of this study are available from the corresponding author upon request.
